# (1*Z*,1′*Z*,3*E*,3′*E*)-1,1′-Diphenyl-3,3′-[(1*S*,2*S*)-cyclo­hexane-1,2-diyldinitrilo]dibut-1-en-1-ol

**DOI:** 10.1107/S1600536808014670

**Published:** 2008-05-21

**Authors:** Xiu-Zhi Li, Zhi-Rong Qu

**Affiliations:** a Ordered Matter Science Research Center, College of Chemistry and Chemical Engineering, Southeast University, Nanjing 210096, People’s Republic of China

## Abstract

A new tetra­dentate chiral Schiff base ligand, C_26_H_30_N_2_O_2_, has been synthesized by the reaction of 1-phenyl­butane-1,3-dione with (1*S*,2*S)*-(−)-1,2-diamino­cyclo­hexane. The chiral centers in the mol­ecule have the same *S* configuration, since the absolute configuration was determined by that of the starting reagent (1*S*,2*S*)-(−)-1,2-diamino­hexane. The cyclo­hexane ring is in a chair conformation, and the substituents are equatorial in the most stable conformation (*trans*-cyclo­hexyl). The crystal structure is stabilized by two intra­molecular O—H⋯N hydrogen bonds and a weak C—H⋯π inter­action.

## Related literature

For the chemistry of Schiff bases, see: Alemi & Shaabani (2000 ▶); Bandini *et al.* (1999 ▶, 2000 ▶); Belokon *et al.* (1997 ▶); Cozzi (2003 ▶); Jiang *et al.* (1995 ▶); Kureshy *et al.* (2001 ▶); Sasaki *et al.* (1991 ▶).
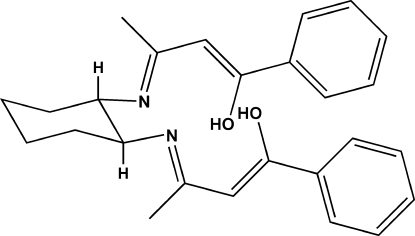


## Experimental

### 

#### Crystal data

C_26_H_30_N_2_O_2_
*M* *_r_* = 402.52
Orthorhombic, 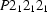

*a* = 8.9073 (11) Å
*b* = 10.1205 (13) Å
*c* = 26.476 (3) Å
*V* = 2386.7 (5) Å^3^
*Z* = 4
Mo *K*α radiation
μ = 0.07 mm^−1^
*T* = 293 (2) K
0.20 × 0.20 × 0.20 mm

#### Data collection

Rigaku SCXmini diffractometer
Absorption correction: multi-scan (*CrystalClear*; Rigaku, 2005 ▶) *T*_min_ = 0.980, *T*_max_ = 0.990
22130 measured reflections
2683 independent reflections
1952 reflections with *I* > 2σ(*I*)
*R*_int_ = 0.062

#### Refinement

*R*[*F* ^2^ > 2σ(*F* ^2^)] = 0.061
*wR*(*F* ^2^) = 0.159
*S* = 1.07
2683 reflections
275 parameters
H-atom parameters constrained
Δρ_max_ = 0.29 e Å^−3^
Δρ_min_ = −0.17 e Å^−3^

### 

Data collection: *CrystalClear* (Rigaku, 2005 ▶); cell refinement: *CrystalClear*; data reduction: *CrystalClear*; program(s) used to solve structure: *SHELXS97* (Sheldrick, 2008 ▶); program(s) used to refine structure: *SHELXL97* (Sheldrick, 2008 ▶); molecular graphics: *SHELXTL* (Sheldrick, 2008 ▶); software used to prepare material for publication: *SHELXL97*.

## Supplementary Material

Crystal structure: contains datablocks I, global. DOI: 10.1107/S1600536808014670/bx2143sup1.cif

Structure factors: contains datablocks I. DOI: 10.1107/S1600536808014670/bx2143Isup2.hkl

Additional supplementary materials:  crystallographic information; 3D view; checkCIF report

## Figures and Tables

**Table 1:** Hydrogen-bond geometry (Å, °)

*D*—H⋯*A*	*D*—H	H⋯*A*	*D*⋯*A*	*D*—H⋯*A*
O2—H2*A*⋯N2	0.82	1.93	2.650 (3)	146
O1—H1⋯N1	0.82	1.91	2.629 (4)	145
C19—H19*A*⋯*Cg*3^i^	0.97	2.96	3.795 (5)	144

Symmetry code: (i) 
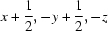
. *Cg*3 is the centroid of the C10–C15 ring.

**Fig. 1.**
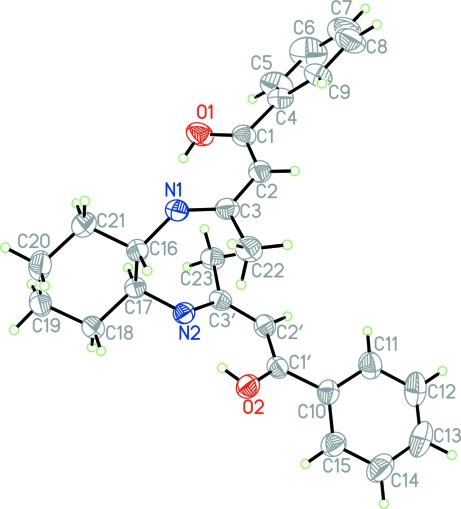
A view of the compound with the atomic numbering scheme. Displacement ellipsoids were drawn at the 30% probability level.

## supplementary crystallographic information

### Comment

In recent years, research on Schiff bases has been intensified for the reason that some of them can form materials with non-linear optical (NLO) activity (Alemi & Shaabani, 2000), and some can be used for the asymmetric oxidation of methyl phenyl sulfides (Sasaki *et al.*, 1991). The search for new chiral ligands for asymmetric synthesis is an important task in organic chemistry. Various chiral Schiff bases are widely used in asymmetric reactions (Jiang *et al.*, 1995; Belokon *et al.*, 1997; Bandini *et al.*, 1999, 2000; Kureshy *et al.*, 2001; Cozzi, 2003). Herein, we report the synthesis and crystal structure of a new chiral Schiff base ligand (1*Z*,1'*Z*,3*E*,3'*E*)-3,3'-((1*S*,2*S*)-cyclohexane-1,2-diylbis(azan-1-yl-1-ylidene))bis(1-phenylbut-1-en-1-ol). Fig. 1 show, the absolute configurations of the chiral centers and they have the same chirality (*S*-configuration). The cyclohexane ring is of chair conformation, and the substituents are equatorial in the most stable conformation of *trans*-cyclohexyl. The crystal structure is stabilized by two intramolecular O—H···N hydrogen bonds and a weak C—H···π interaction (Table 1).

### Experimental

1-phenylbutane-1,3-dione (3.89 g, 0.024 mol) in 6 ml of chloroform was added dropwise to a solution of chloroform (20 ml) containing (1*S*, 2*S*)-(–)-1,2-diaminocyclohexane (1.14 g, 0.01 mol), which was kept at 0–5°C with vigorous stirring during the reaction. After complete addition which took approximately 30 min, the mixture was stirred for another 1 h at room temperature. After the evaporation of the solvent under reduced pressure, the crude product was recrystallized by slowly evaporating with petroleum ether to yield colorless crystals.

### Refinement

Positional parameters of all the H atoms were calculated geometrically and were allowed to ride on the C, O atoms to which they are bonded, with C—H = 0.93 to 0.98Å, O—H = 0.82 Å , with U_iso_ (H) = 1.2U_eq_ (C_aromatic_, C_methylene_), U_iso_(H) = 1.5U_eq_ (C_methyl_) or 1.5 U_eq_(O). In the absence of significant anomalous scattering effects, 2006 Friedel pairs were merged.

### Crystal data

**Table:**

C_26_H_30_N_2_O_2_	*F*_000_ = 864
*M**_r_* = 402.52	*D*_x_ = 1.120 Mg m^−^^3^
Orthorhombic, *P*2_1_2_1_2_1_	Mo *K*α radiation λ = 0.71073 Å
Hall symbol: P 2ac 2ab	Cell parameters from 4136 reflections
*a* = 8.9073 (11) Å	θ = 3.1–27.5º
*b* = 10.1205 (13) Å	µ = 0.07 mm^−^^1^
*c* = 26.476 (3) Å	*T* = 293 (2) K
*V* = 2386.7 (5) Å^3^	Block, colorless
*Z* = 4	0.20 × 0.20 × 0.20 mm

### Data collection

**Table:**

Rigaku SCXmini diffractometer	2683 independent reflections
Radiation source: fine-focus sealed tube	1952 reflections with *I* > 2σ(*I*)
Monochromator: graphite	*R*_int_ = 0.062
Detector resolution: 13.6612 pixels mm^-1^	θ_max_ = 26.0º
*T* = 293(2) K	θ_min_ = 2.4º
ω scans	*h* = −10→10
Absorption correction: multi-scan(CrystalClear; Rigaku, 2005)	*k* = −12→12
*T*_min_ = 0.980, *T*_max_ = 0.990	*l* = −32→32
22130 measured reflections	

### Refinement

**Table:**

Refinement on *F*^2^	Secondary atom site location: difference Fourier map
Least-squares matrix: full	Hydrogen site location: inferred from neighbouring sites
*R*[*F*^2^ > 2σ(*F*^2^)] = 0.061	H-atom parameters constrained
*wR*(*F*^2^) = 0.159	*w* = 1/[σ^2^(*F*_o_^2^) + (0.0753*P*)^2^ + 0.2595*P*] where *P* = (*F*_o_^2^ + 2*F*_c_^2^)/3
*S* = 1.07	(Δ/σ)_max_ < 0.001
2683 reflections	Δρ_max_ = 0.29 e Å^−^^3^
275 parameters	Δρ_min_ = −0.17 e Å^−^^3^
Primary atom site location: structure-invariant direct methods	Extinction correction: none

### Special details

**Table:**

Geometry. All e.s.d.'s (except the e.s.d. in the dihedral angle between two l.s. planes) are estimated using the full covariance matrix. The cell e.s.d.'s are taken into account individually in the estimation of e.s.d.'s in distances, angles and torsion angles; correlations between e.s.d.'s in cell parameters are only used when they are defined by crystal symmetry. An approximate (isotropic) treatment of cell e.s.d.'s is used for estimating e.s.d.'s involving l.s. planes.
Refinement. Refinement of *F*^2^ against ALL reflections. The weighted *R*-factor *wR* and goodness of fit *S* are based on *F*^2^, conventional *R*-factors *R* are based on *F*, with *F* set to zero for negative *F*^2^. The threshold expression of *F*^2^ > σ(*F*^2^) is used only for calculating *R*-factors(gt) *etc*. and is not relevant to the choice of reflections for refinement. *R*-factors based on *F*^2^ are statistically about twice as large as those based on *F*, and *R*- factors based on ALL data will be even larger.

### Fractional atomic coordinates and isotropic or equivalent isotropic displacement parameters (Å^2^)

**Table:**

	*x*	*y*	*z*	*U*_iso_*/*U*_eq_	
N2	0.8242 (3)	0.2435 (3)	0.96116 (10)	0.0576 (7)	
C17	0.8209 (4)	0.1345 (3)	0.92486 (12)	0.0562 (8)	
H17	0.9072	0.1434	0.9021	0.067*	
N1	0.6697 (4)	0.2620 (3)	0.86429 (10)	0.0618 (8)	
O2	0.7103 (3)	0.3353 (2)	1.04671 (9)	0.0684 (7)	
H2A	0.7189	0.2838	1.0230	0.103*	
C1'	0.7786 (4)	0.4406 (3)	1.03675 (13)	0.0582 (8)	
C18	0.8342 (5)	0.0039 (3)	0.95283 (14)	0.0669 (10)	
H18A	0.9290	0.0014	0.9708	0.080*	
H18B	0.7541	−0.0025	0.9776	0.080*	
C2	0.6168 (4)	0.4861 (4)	0.84749 (13)	0.0597 (9)	
H2	0.5630	0.5613	0.8564	0.072*	
C3'	0.8907 (4)	0.3615 (4)	0.95676 (13)	0.0571 (8)	
C3	0.5973 (4)	0.3737 (4)	0.87611 (13)	0.0597 (9)	
C21	0.6703 (6)	0.0219 (4)	0.85724 (15)	0.0776 (12)	
H21A	0.5761	0.0238	0.8389	0.093*	
H21B	0.7512	0.0291	0.8329	0.093*	
O1	0.7936 (4)	0.3983 (3)	0.79076 (10)	0.0852 (9)	
H1	0.7875	0.3376	0.8112	0.128*	
C16	0.6767 (4)	0.1386 (3)	0.89317 (13)	0.0584 (9)	
H16	0.5899	0.1345	0.9159	0.070*	
C4	0.7242 (4)	0.6191 (4)	0.77629 (13)	0.0653 (10)	
C2'	0.8698 (4)	0.4569 (4)	0.99355 (13)	0.0618 (9)	
H2'	0.9192	0.5372	0.9896	0.074*	
C10	0.7610 (4)	0.5522 (4)	1.07347 (14)	0.0648 (9)	
C23	0.9890 (5)	0.3907 (4)	0.91182 (14)	0.0684 (10)	
H23A	0.9330	0.3765	0.8813	0.103*	
H23B	1.0747	0.3332	0.9123	0.103*	
H23C	1.0219	0.4809	0.9132	0.103*	
C1	0.7137 (4)	0.4938 (4)	0.80549 (13)	0.0629 (9)	
C22	0.4918 (5)	0.3766 (4)	0.92075 (15)	0.0778 (12)	
H22A	0.5438	0.3469	0.9504	0.117*	
H22B	0.4079	0.3194	0.9143	0.117*	
H22C	0.4565	0.4652	0.9259	0.117*	
C19	0.8253 (5)	−0.1133 (4)	0.91717 (15)	0.0772 (11)	
H19A	0.9126	−0.1136	0.8953	0.093*	
H19B	0.8262	−0.1946	0.9366	0.093*	
C20	0.6840 (6)	−0.1081 (4)	0.88527 (18)	0.0847 (12)	
H20A	0.6855	−0.1801	0.8611	0.102*	
H20B	0.5971	−0.1197	0.9069	0.102*	
C15	0.7371 (5)	0.5242 (4)	1.12405 (15)	0.0771 (11)	
H15	0.7328	0.4368	1.1348	0.093*	
C9	0.6005 (5)	0.6985 (4)	0.76822 (16)	0.0787 (12)	
H9	0.5080	0.6744	0.7817	0.094*	
C11	0.7644 (5)	0.6836 (4)	1.05852 (18)	0.0824 (13)	
H11	0.7793	0.7047	1.0247	0.099*	
C12	0.7458 (6)	0.7824 (5)	1.0933 (2)	0.1040 (17)	
H12	0.7483	0.8701	1.0828	0.125*	
C8	0.6128 (7)	0.8127 (5)	0.74050 (19)	0.1055 (17)	
H8	0.5290	0.8658	0.7352	0.127*	
C14	0.7197 (7)	0.6255 (5)	1.15854 (18)	0.1006 (16)	
H14	0.7049	0.6065	1.1925	0.121*	
C5	0.8588 (5)	0.6560 (6)	0.75543 (18)	0.1002 (16)	
H5	0.9425	0.6022	0.7597	0.120*	
C13	0.7242 (6)	0.7548 (6)	1.1424 (2)	0.1062 (18)	
H13	0.7123	0.8232	1.1655	0.127*	
C6	0.8718 (8)	0.7720 (7)	0.7282 (3)	0.133 (2)	
H6	0.9640	0.7979	0.7150	0.160*	
C7	0.7471 (11)	0.8477 (6)	0.7210 (2)	0.126 (2)	
H7	0.7546	0.9251	0.7023	0.152*	

### Atomic displacement parameters (Å^2^)

**Table:**

	*U*^11^	*U*^22^	*U*^33^	*U*^12^	*U*^13^	*U*^23^
N2	0.0638 (17)	0.0557 (16)	0.0532 (15)	−0.0045 (15)	0.0003 (14)	−0.0004 (14)
C17	0.061 (2)	0.0553 (19)	0.0528 (17)	0.0016 (18)	0.0041 (16)	0.0009 (16)
N1	0.0690 (19)	0.0633 (18)	0.0531 (15)	0.0081 (17)	−0.0017 (15)	0.0041 (14)
O2	0.0813 (18)	0.0624 (15)	0.0614 (14)	−0.0075 (15)	0.0111 (14)	−0.0046 (12)
C1'	0.062 (2)	0.0504 (18)	0.0618 (19)	−0.0020 (18)	−0.0071 (18)	0.0042 (17)
C18	0.077 (3)	0.059 (2)	0.065 (2)	0.003 (2)	−0.006 (2)	0.0059 (18)
C2	0.063 (2)	0.061 (2)	0.0551 (18)	0.0043 (18)	0.0048 (17)	0.0030 (17)
C3'	0.0552 (19)	0.0579 (19)	0.0582 (19)	−0.0051 (17)	−0.0012 (17)	0.0089 (17)
C3	0.059 (2)	0.070 (2)	0.0505 (18)	0.006 (2)	0.0025 (16)	0.0044 (18)
C21	0.091 (3)	0.073 (3)	0.069 (2)	−0.002 (2)	−0.013 (2)	−0.011 (2)
O1	0.091 (2)	0.097 (2)	0.0675 (16)	0.0300 (19)	0.0233 (16)	0.0236 (15)
C16	0.065 (2)	0.0539 (19)	0.0566 (18)	0.0023 (19)	0.0052 (18)	0.0011 (16)
C4	0.065 (2)	0.077 (2)	0.0537 (18)	−0.010 (2)	−0.0035 (18)	0.0070 (19)
C2'	0.067 (2)	0.0527 (19)	0.066 (2)	−0.0099 (18)	0.0021 (18)	0.0005 (18)
C10	0.061 (2)	0.061 (2)	0.072 (2)	0.0020 (19)	−0.0050 (19)	−0.0072 (19)
C23	0.067 (2)	0.072 (2)	0.066 (2)	−0.007 (2)	0.0063 (19)	0.008 (2)
C1	0.058 (2)	0.074 (2)	0.0565 (18)	0.009 (2)	0.0001 (17)	0.0050 (19)
C22	0.080 (3)	0.083 (3)	0.070 (2)	0.014 (2)	0.026 (2)	0.014 (2)
C19	0.090 (3)	0.056 (2)	0.086 (3)	0.006 (2)	0.004 (2)	0.001 (2)
C20	0.100 (3)	0.060 (2)	0.094 (3)	−0.001 (2)	−0.003 (3)	−0.015 (2)
C15	0.082 (3)	0.083 (3)	0.067 (2)	0.004 (2)	−0.005 (2)	−0.008 (2)
C9	0.078 (3)	0.083 (3)	0.076 (3)	0.001 (2)	−0.012 (2)	0.020 (2)
C11	0.088 (3)	0.064 (2)	0.095 (3)	0.000 (2)	0.007 (3)	−0.001 (2)
C12	0.106 (4)	0.064 (3)	0.142 (5)	0.002 (3)	0.016 (4)	−0.023 (3)
C8	0.136 (5)	0.090 (3)	0.090 (3)	−0.001 (4)	−0.018 (3)	0.031 (3)
C14	0.110 (4)	0.114 (4)	0.078 (3)	0.014 (4)	−0.010 (3)	−0.030 (3)
C5	0.077 (3)	0.124 (4)	0.100 (3)	−0.017 (3)	0.012 (3)	0.031 (3)
C13	0.096 (4)	0.091 (4)	0.131 (5)	0.001 (3)	0.001 (4)	−0.054 (4)
C6	0.120 (5)	0.149 (6)	0.130 (5)	−0.056 (5)	0.021 (4)	0.039 (5)
C7	0.180 (7)	0.099 (4)	0.100 (4)	−0.037 (5)	−0.006 (5)	0.034 (3)

### Geometric parameters (Å, °)

**Table:**

N2—C3'	1.338 (4)	C10—C15	1.385 (5)
N2—C17	1.463 (4)	C10—C11	1.388 (5)
C17—C18	1.520 (5)	C23—H23A	0.9600
C17—C16	1.535 (5)	C23—H23B	0.9600
C17—H17	0.9800	C23—H23C	0.9600
N1—C3	1.339 (5)	C22—H22A	0.9600
N1—C16	1.465 (4)	C22—H22B	0.9600
O2—C1'	1.255 (4)	C22—H22C	0.9600
O2—H2A	0.8200	C19—C20	1.517 (6)
C1'—C2'	1.413 (5)	C19—H19A	0.9700
C1'—C10	1.498 (5)	C19—H19B	0.9700
C18—C19	1.518 (5)	C20—H20A	0.9700
C18—H18A	0.9700	C20—H20B	0.9700
C18—H18B	0.9700	C15—C14	1.382 (6)
C2—C3	1.378 (5)	C15—H15	0.9300
C2—C1	1.409 (5)	C9—C8	1.374 (6)
C2—H2	0.9300	C9—H9	0.9300
C3'—C2'	1.384 (5)	C11—C12	1.370 (6)
C3'—C23	1.506 (5)	C11—H11	0.9300
C3—C22	1.510 (5)	C12—C13	1.341 (7)
C21—C20	1.515 (5)	C12—H12	0.9300
C21—C16	1.517 (5)	C8—C7	1.350 (8)
C21—H21A	0.9700	C8—H8	0.9300
C21—H21B	0.9700	C14—C13	1.377 (7)
O1—C1	1.262 (4)	C14—H14	0.9300
O1—H1	0.8200	C5—C6	1.382 (8)
C16—H16	0.9800	C5—H5	0.9300
C4—C5	1.372 (6)	C13—H13	0.9300
C4—C9	1.380 (5)	C6—C7	1.363 (9)
C4—C1	1.488 (5)	C6—H6	0.9300
C2'—H2'	0.9300	C7—H7	0.9300
			
C3'—N2—C17	128.6 (3)	H23A—C23—H23C	109.5
N2—C17—C18	109.5 (3)	H23B—C23—H23C	109.5
N2—C17—C16	110.8 (3)	O1—C1—C2	123.2 (3)
C18—C17—C16	110.8 (3)	O1—C1—C4	117.2 (3)
N2—C17—H17	108.6	C2—C1—C4	119.7 (3)
C18—C17—H17	108.6	C3—C22—H22A	109.5
C16—C17—H17	108.6	C3—C22—H22B	109.5
C3—N1—C16	128.2 (3)	H22A—C22—H22B	109.5
C1'—O2—H2A	109.5	C3—C22—H22C	109.5
O2—C1'—C2'	123.2 (3)	H22A—C22—H22C	109.5
O2—C1'—C10	116.9 (3)	H22B—C22—H22C	109.5
C2'—C1'—C10	119.8 (3)	C20—C19—C18	111.2 (3)
C19—C18—C17	111.9 (3)	C20—C19—H19A	109.4
C19—C18—H18A	109.2	C18—C19—H19A	109.4
C17—C18—H18A	109.2	C20—C19—H19B	109.4
C19—C18—H18B	109.2	C18—C19—H19B	109.4
C17—C18—H18B	109.2	H19A—C19—H19B	108.0
H18A—C18—H18B	107.9	C21—C20—C19	111.7 (4)
C3—C2—C1	123.8 (3)	C21—C20—H20A	109.3
C3—C2—H2	118.1	C19—C20—H20A	109.3
C1—C2—H2	118.1	C21—C20—H20B	109.3
N2—C3'—C2'	120.1 (3)	C19—C20—H20B	109.3
N2—C3'—C23	120.1 (3)	H20A—C20—H20B	107.9
C2'—C3'—C23	119.8 (3)	C14—C15—C10	120.3 (4)
N1—C3—C2	120.5 (3)	C14—C15—H15	119.9
N1—C3—C22	119.9 (3)	C10—C15—H15	119.9
C2—C3—C22	119.6 (3)	C8—C9—C4	120.6 (5)
C20—C21—C16	111.5 (3)	C8—C9—H9	119.7
C20—C21—H21A	109.3	C4—C9—H9	119.7
C16—C21—H21A	109.3	C12—C11—C10	120.3 (5)
C20—C21—H21B	109.3	C12—C11—H11	119.8
C16—C21—H21B	109.3	C10—C11—H11	119.8
H21A—C21—H21B	108.0	C13—C12—C11	121.1 (5)
C1—O1—H1	109.5	C13—C12—H12	119.4
N1—C16—C21	109.5 (3)	C11—C12—H12	119.4
N1—C16—C17	110.1 (3)	C7—C8—C9	119.7 (6)
C21—C16—C17	110.7 (3)	C7—C8—H8	120.2
N1—C16—H16	108.8	C9—C8—H8	120.2
C21—C16—H16	108.8	C13—C14—C15	119.8 (5)
C17—C16—H16	108.8	C13—C14—H14	120.1
C5—C4—C9	118.5 (4)	C15—C14—H14	120.1
C5—C4—C1	119.7 (4)	C4—C5—C6	120.9 (6)
C9—C4—C1	121.8 (4)	C4—C5—H5	119.5
C3'—C2'—C1'	124.5 (3)	C6—C5—H5	119.5
C3'—C2'—H2'	117.8	C12—C13—C14	120.2 (5)
C1'—C2'—H2'	117.8	C12—C13—H13	119.9
C15—C10—C11	118.3 (4)	C14—C13—H13	119.9
C15—C10—C1'	119.3 (3)	C7—C6—C5	118.8 (6)
C11—C10—C1'	122.3 (4)	C7—C6—H6	120.6
C3'—C23—H23A	109.5	C5—C6—H6	120.6
C3'—C23—H23B	109.5	C8—C7—C6	121.5 (5)
H23A—C23—H23B	109.5	C8—C7—H7	119.3
C3'—C23—H23C	109.5	C6—C7—H7	119.3
			
C3'—N2—C17—C18	141.4 (4)	C3—C2—C1—O1	−1.2 (6)
C3'—N2—C17—C16	−96.1 (4)	C3—C2—C1—C4	178.6 (3)
N2—C17—C18—C19	177.8 (3)	C5—C4—C1—O1	−34.7 (6)
C16—C17—C18—C19	55.2 (4)	C9—C4—C1—O1	143.5 (4)
C17—N2—C3'—C2'	174.1 (3)	C5—C4—C1—C2	145.5 (4)
C17—N2—C3'—C23	−6.1 (5)	C9—C4—C1—C2	−36.3 (5)
C16—N1—C3—C2	172.3 (3)	C17—C18—C19—C20	−54.6 (5)
C16—N1—C3—C22	−8.4 (6)	C16—C21—C20—C19	−55.7 (5)
C1—C2—C3—N1	−1.4 (6)	C18—C19—C20—C21	54.4 (5)
C1—C2—C3—C22	179.4 (4)	C11—C10—C15—C14	−1.0 (7)
C3—N1—C16—C21	141.4 (4)	C1'—C10—C15—C14	−179.6 (4)
C3—N1—C16—C17	−96.7 (4)	C5—C4—C9—C8	−0.9 (6)
C20—C21—C16—N1	177.5 (4)	C1—C4—C9—C8	−179.1 (4)
C20—C21—C16—C17	56.0 (5)	C15—C10—C11—C12	0.6 (7)
N2—C17—C16—N1	61.4 (3)	C1'—C10—C11—C12	179.1 (4)
C18—C17—C16—N1	−176.9 (3)	C10—C11—C12—C13	0.0 (9)
N2—C17—C16—C21	−177.4 (3)	C4—C9—C8—C7	0.0 (7)
C18—C17—C16—C21	−55.6 (4)	C10—C15—C14—C13	0.8 (8)
N2—C3'—C2'—C1'	−1.1 (6)	C9—C4—C5—C6	1.9 (7)
C23—C3'—C2'—C1'	179.1 (3)	C1—C4—C5—C6	−179.9 (5)
O2—C1'—C2'—C3'	2.2 (6)	C11—C12—C13—C14	−0.3 (9)
C10—C1'—C2'—C3'	−178.4 (3)	C15—C14—C13—C12	−0.2 (9)
O2—C1'—C10—C15	30.0 (5)	C4—C5—C6—C7	−2.0 (9)
C2'—C1'—C10—C15	−149.4 (4)	C9—C8—C7—C6	0.0 (10)
O2—C1'—C10—C11	−148.5 (4)	C5—C6—C7—C8	1.0 (11)
C2'—C1'—C10—C11	32.1 (6)		

### Hydrogen-bond geometry (Å, °)

**Table:**

*D*—H···*A*	*D*—H	H···*A*	*D*···*A*	*D*—H···*A*
O2—H2A···N2	0.82	1.93	2.650 (3)	146
O1—H1···N1	0.82	1.91	2.629 (4)	145
C19—H19A···Cg3^i^	0.97	2.96	3.795 (5)	144

Symmetry codes: (i) *x*+1/2, −*y*+1/2, −*z*.
